# Clemastine ameliorates ulcerative colitis-induced cognitive impairment by restoring PI3K/Akt/GSK-3β signaling and suppressing ferroptosis

**DOI:** 10.1007/s10787-026-02303-5

**Published:** 2026-06-30

**Authors:** Manar A. Didamoony, Mustafa M. Shokr, Reem M. Eladawy, Lamiaa A. Ahmed

**Affiliations:** 1 Pharmacology and Toxicology Department, Faculty of Pharmacy, Egyptian Russian University, Cairo, 11829 Egypt; 2 Department of Pharmacology and Toxicology, Faculty of Pharmacy, Sinai University—Arish Branch, Arish, 45511 Egypt; 3 Pharmacology and Toxicology Department, Faculty of Pharmacy, Cairo University, Cairo, 11562 Egypt

**Keywords:** Autophagy, Clemastine, Ferroptosis, Neuroinflammation, Ulcerative colitis

## Abstract

Ulcerative colitis (UC) is a refractory inflammatory bowel disease with ongoing colonic inflammation and extra-intestinal manifestations, including cognitive impairment. In this study, we evaluated the peripheral and central effects of clemastine on cognitive impairment induced by UC using an acetic acid (1 ml, 4% v/v) model with emphasis on the role of PI3K/Akt/GSK-3β signaling, ferroptosis, and autophagy in its possible mediated neuroprotection. Clemastine revealed a dose-dependent improvement of colonic damage linked with UC, as demonstrated by inhibition of TNF-α, IL-1β, and caspase-3 levels along with upregulation of claudin-1 expression. Importantly, the preservation of gut membrane integrity was associated with amelioration of UC-induced systemic inflammation together with restoration of PI3K/Akt/GSK-3β signaling in the brain. Additionally, the neuroprotective actions of clemastine included the prevention of neuronal injury and death as evidenced by inhibition of oxidative stress markers and microglial activation (Iba-1 expression), which could exacerbate the neuroinflammatory response. Concurrently, inhibition of the pathological autophagic dysfunction and ferroptosis profile by clemastine, as clarified by LC3-II downregulation and LC3-I and GPX4 upregulation, led to behavioral and cognitive improvements, which were proven by decreased levels of Aβ and tau protein. Importantly, by crossing the BBB, clemastine could exert additional anti-inflammatory and neuroprotective effects by affecting the central assessed pathways. In conclusion, these findings clarify the pivotal role of clemastine in the management of colonic inflammation-associated cognitive decline through modulating multiple and interacting peripheral and central pathways to provide its potent neuroprotection.

## Introduction

Ulcerative colitis (UC) is a chronic inflammatory bowel disease characterized by persistent mucosal inflammation of the colon with accompanying symptoms of bloody diarrhea, abdominal pain, and weight loss (Danese et al. 2025). In addition to gastrointestinal symptoms, recent studies increasingly suggest evidence of an existing connection between UC and extra-intestinal complications, especially those affecting the central nervous system (CNS), including cognitive decline (Kim et al. 2023; Elbaz et al. 2023).

UC is characterized by peripheral colonic inflammation induced by elevated levels of pro-inflammatory mediators, including tumor necrosis factor-alpha (TNF-α) and interleukin 1 beta (IL-1β), leading to different manifestations in the gut lining membrane. These changes encompass the induction of apoptosis with downregulation of important gut junction proteins such as claudin-1 (Bhol et al. 2024). This cascade is continued with a status of systemic inflammation, which is the major proposed mechanism for inducing neuroinflammation and potentially neurodegeneration in patients with UC (Bhol et al. 2024). Elevated pro-inflammatory cytokines, including TNF-α has been known to be associated with impaired blood-brain barrier (BBB) in a manner that prompts a neuroinflammatory response (Adamu et al. 2024; Bhol et al. 2024). TNF-α ‘s role in neurodegeneration has been suggested to affect intracellular signaling pathways, which are critical for neuronal survival and synaptic plasticity (Gonzalez Caldito 2023). One of the aforementioned pathways includes the phosphatidylinositol-3-kinase (PI3K)/phosphokinase B (Akt)/glycogen synthase kinase-3beta (GSK-3β) axis (Gogulescu et al. 2024). Dysregulation of this pathway, which is important in cell survival, metabolism, and cell proliferation, is a prominent feature of multiple neurodegenerative diseases (Razani et al. 2021; Wright et al. 2024). Deficits in PI3K/Akt activation by TNF-α-induced neuroinflammation decrease GSK-3β phosphorylation and inactivation (Limantoro et al. 2023), which is detrimental due to the induction of ferroptosis, a form of iron-dependent cell death, with increased levels of acyl-CoA synthetase long chain family member 4 (ACSL4) and decreased levels of glutathione peroxidase 4 (GPX4).

Inhibition of autophagy and induction of oxidative stress, other outcomes of the activated GSK-3β, may result in additional neurotoxicity, decreased neurogenesis, and cognitive decline due to elevated levels of amyloid beta (Aβ) and tau protein phosphorylation (Limantoro et al. 2023; Muro et al. 2024). Therefore, UC may mediate neuroinflammation and neurodegeneration through a complex interaction of signaling cascades activated by systemic inflammatory cytokines, including TNF-α/PI3K/Akt/GSK-3β axis, suggesting a link between cognitive decline and chronic gut inflammation (Sun et al. 2022).

Clemastine, a first-generation antihistamine, has been increasingly recognized for its possible therapeutic potential, especially in neurological settings. Clemastine seems to have some modulatory effects that increase oligodendrocyte differentiation and remyelination, two key elements involved in the maintenance of white matter integrity and cognitive function (Jiang et al. 2023a). The potential for clemastine to promote remyelination suggests its future value in limiting cognitive decline associated with conditions related to acute and chronic CNS inflammation (Yamazaki and Ohno 2025). Clemastine also exerts anti-inflammatory properties, including but not limited to the inhibition of neuroinflammation associated with microglial activation (Su et al. 2018). Therefore, the current study aimed to investigate the effect of clemastine in mitigating the cognitive impairment induced by UC in rats. More specifically, the present work examined the peripheral and central effects of clemastine, including TNF-α/PI3K/Akt/GSK-3β axis, in this possible mediated neuroprotection.

## Materials and methods

### Animals

In this investigation, fifty Sprague Dawley male rats weighing between 200 and 250 g were used. The National Research Centre in Cairo, Egypt, is the place from which animals were bought. During the experiment, rats were maintained on a typical pellet diet and tap water, with a 12-hour light-dark cycle and an average temperature of 22–25 °C. The Ethical Guidelines for Experimental Research at Research Ethics Committee [Faculty of Pharmacy, Sinai University, Approval No. (2025), (83 A)] and the Guide for Care and Use of Experimental Animals [NIH Publication No. 80 − 23, revised 2011] were closely followed in the management of all experimental procedures.

### Drug preparation

Clemastine and acetic acid were obtained from Novartis (Cairo, Egypt) and El-Nasr Pharmaceutical Co., Egypt, respectively, and were dissolved in 0.9% saline. All drugs and chemicals were freshly prepared before being administered.

### Experimental design

Fifty rats were weighed and divided randomly into five groups (*n* = 10 per group) as follows:

*Group 1 (normal group)* rats were given a single intrarectal dose of 1 ml saline (0.9%) on the first day of the experiment and daily saline (0.9%) orally equivalent to the drug volume for 14 days.

*Group 2 (acetic acid group)* rats were given a single dose of intrarectal acetic acid (1 ml, 4%, v/v) for UC induction on the first day of the experiment (Elbaz et al. 2023).

*Group 3 (acetic acid + clemastine 5 mg)* rats were given a single dose of intrarectal acetic acid (1 ml, 4%, v/v) on the first day of the experiment, followed by clemastine orally (5 mg/kg/day) for 14 days (Motawi et al. 2023).

*Group 4 (acetic acid + clemastine 7.5 mg)* rats were given a single dose of intrarectal acetic acid (1 ml, 4%, v/v) on the first day of the experiment, followed by clemastine orally (7.5 mg/kg/day) for 14 days (Odell et al. 2024).

**Fig. 1:**
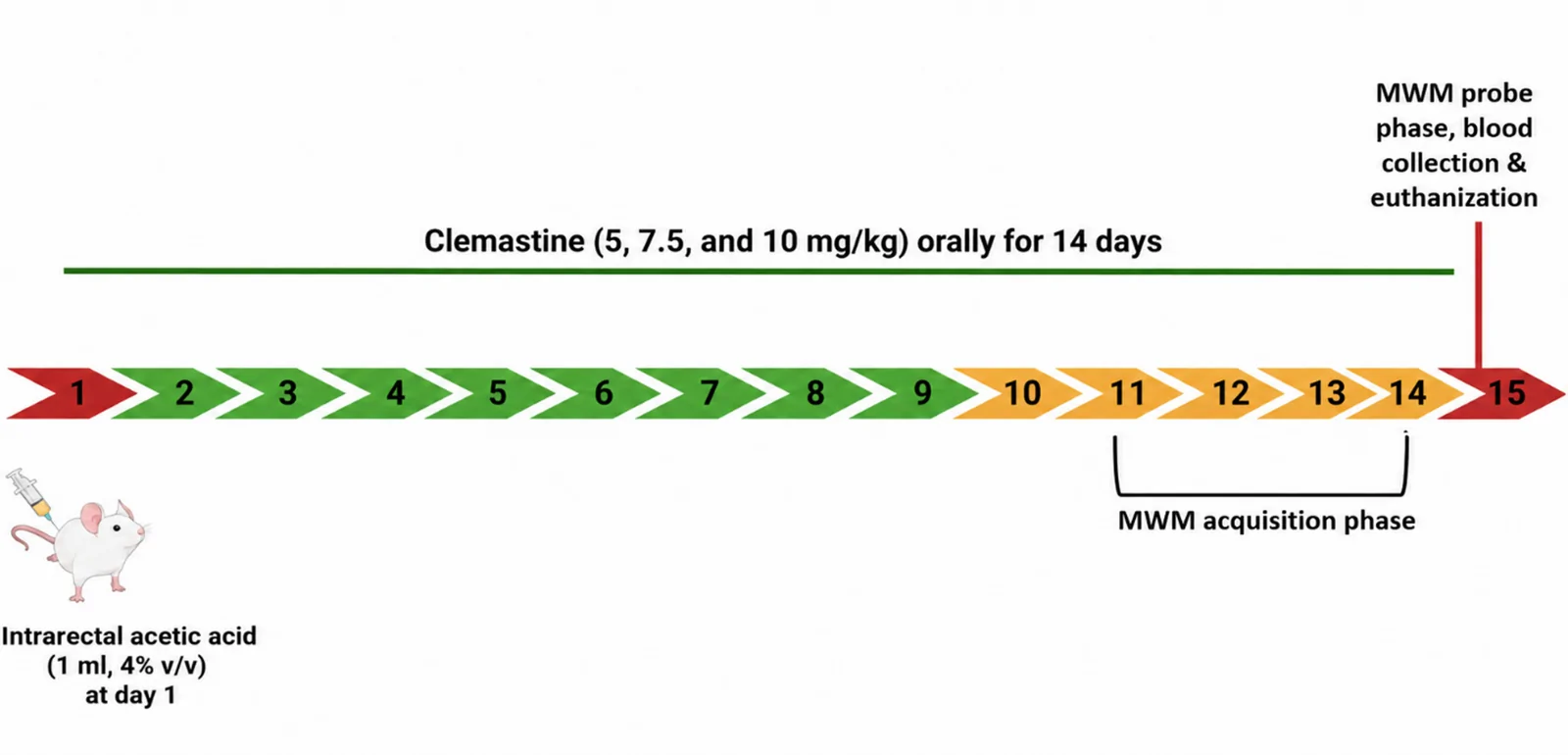
Schematic diagram of the experimental design

*Group 5 (acetic acid + clemastine 10 mg)* rats were given a single dose of intrarectal acetic acid (1 ml, 4%, v/v) on the first day of the experiment, followed by clemastine orally (10 mg/kg/day) for 14 days (Badawi et al. 2024) (Fig. 1).

The doses of clemastine (5, 7.5, and 10 mg/kg/day) were selected based on previous experimental studies demonstrating the neuroprotective effects of clemastine in autoimmune encephalomyelitis (Motawi et al. 2023), PTZ-induced kindling (Badawi et al. 2024), and preterm white matter injury (Odell et al. 2024) without observable sedative effects or motor impairment, allowing the robust evaluation of these doses in our model. Importantly, in the present work, all the behavioral testing, histopathological assessments, and biochemical analyses were performed by specialists blinded to the treatment groups. Additionally, group allocation codes were maintained by an independent investigator until completion of all data acquisition and statistical analysis.

### Induction of UC

On the first day of the experiment, acetic acid was used to produce UC using a technique previously reported by Millar et al. (1996). Rats were given full access to water for 24-hour during the fasting time before being anesthetized with intraperitoneal thiopental (20 mg/kg) (Elbaz et al. 2023). Using a polyethylene tube (2 mm in diameter), a single intra-rectal dosage of acetic acid (1 ml, 4%, v/v) was administered to each rat. The tube was placed 8 cm proximal to the anus. To prevent leakage of acetic acid, the animals were turned upside down for a minute before being returned to their cages.

### Behavioral assay

#### Morris water maze (MWM) test

Using the MWM test, the effect of clemastine on the learning and memory deficits was assessed on the 11th day of the experiment by an expert researcher who was unaware of the experimental groups. A circular escape platform, measuring 15 cm in diameter and 30 cm in height, was submerged approximately 1 cm beneath a layer of ink-stained water in a pool that was 170 cm in diameter and 60 cm in height, evenly divided into four quadrants, and filled with water (22–25 °C). During the first (acquisition) phase, which lasted for four successive days (from day 11 to day 14), each rat was trained for 120 s and allowed to swim freely in an attempt to find the hidden platform in the target quadrant. After being on the platform within 2 min, each rat could stay there for 10 s; if it didn’t reach platform within 2 min, it had to stay for 30 s. On every day of the acquisition phase, the escape latency was appraised where successful learning was demonstrated by lowering of escape latency throughout acquisition sessions compared with the initial session. In the second (probe) phase (day 15), the escape platform was removed and each animal was placed in the pool on the opposite side of the target quadrant and allowed to explore freely for 60 s. Time spent in the target quadrant was quantified using an overhead camera and movements of the rats were tracked with ANY-maze Behavioural tracking software, version 9 (Stoelting Co, IL, USA) (Elbaz et al. 2023; Nasser et al. 2025).

### Sample preparation

Following the behavioral assessment and 24 h after the last dose of treatment, the anesthetized rats with thiopental (40 mg/kg/i.p) were euthanized by cervical dislocation after collection of blood from their retro-orbital plexus (Khodir et al. 2019). For the biochemical assessments, the brains of six rats were used per group, where their hippocampi were carefully removed. Parts of hippocampal tissues were homogenized and processed, where supernatants were collected and stored at − 80 °C until the ELISA evaluation of PI3K, AKT, GSK-3β, nuclear factor -related factor 2 (Nrf2), LC3-I, and LC3-II. Furthermore, other hippocampal tissues were used for colorimetric assessment of malondialdehyde (MDA) and RT-PCR evaluation of ASCL-4 and GPX4. The other four rats’ brains per group were fixed in 10% formaldehyde for histopathological and immunohistochemical analyses.

For colon sampling, the distal colon of each rat was removed, cleaned, and a portion from 0.5 to 1 cm in length of this removed colon (*n* = 4 per group) was preserved in 10% formalin and fixed in paraffin for immunohistochemical and histological examination. Using a polytron homogenizer (Tri-R Stir-R homogenizer, Tri-R Instruments, Inc., Rockville Centre, NY), the remaining colonic tissues from other rats (*n* = 6 per group) were homogenized. The supernatants were separated by centrifugation and kept at -80 °C until the biochemical analysis of TNF-α and caspase-3.

### Brain and colon histopathological analyses

All colon and brain samples were washed with water, dehydrated using a series of alcohol dilutions, cleaned with xylene, and then soaked in paraffin for a day. The paraffinized blocks were cut into 4 μm thick sections, which were subsequently stained with hematoxylin and eosin. The light microscope (BX43, Olympus; Tokyo, Japan) was used in a blinded way to evaluate 2 non-overlapping observational fields per rat in colonic and brain sections to assess colonic and hippocampal abnormalities (Bancroft and Gamble 2008).

### Immunohistochemical analysis

IHC analysis of claudin-1 was assessed in colonic sections, while ionized calcium-binding adapter molecule 1 (Iba1), Aβ and tau protein were evaluated in brain sections. Brain and colonic sections, measuring 4–5 μm, were added to positively charged slides, deparaffinized, rehydrated, and then heat-induced epitope retrieval procedures were applied. Overnight at 4 °C in the refrigerator, colon slides from each group were incubated with anti-claudin-1 (1:100) (catalogue no. 2H10D1, monoclonal antibody, ThermoFisher Scientific Co.). Hippocampal samples were incubated with anti-Ionized calcium-binding adapter molecule 1 (Iba1) (1:10000) (catalogue no. A27316, monoclonal antibody, ABclonal Co.), anti-Aβ (1:600) (catalogue no. GB11307, polyclonal antibody, Servicebio Co.), and anti-tau protein (1:100) (catalogue no. A1103, polyclonal antibody, ABclonal Co.). Following these procedures, the slides were incubated for two hours with a goat anti-mouse secondary antibody (Abcam, Cambridge, UK) with horseradish peroxidase label. The immunoreactivity detection period was carried out using a diaminobenzidine-substrate kit (Thermo Scientific). Area percentage of expression as measured by image J (version 1.53 t, USA)in 2 randomly selected, non-overlapping fields from each sample was used to evaluate and analyze positive staining (Yang et al. 2004).

### Enzyme-linked immunosorbent assays (ELISA)

In compliance with the manufacturers’ instructions, the prepared supernatants were used for assessments of the following parameters: serum and colonic TNF-α (catalogue no. SEA133Ra, Cloud-Clone corp, USA), colonic caspase-3 (catalogue no. SEA626Ra, Cloud-Clone corp, USA), hippocampal PI3K (catalogue no. MBS260381, MyBioSource, USA), hippocampal AKT (catalogue no. LS-F49321, LifeSpan BioSciences Inc, USA), hippocampal GSK-3β (catalogue no. ER0060, Wuhan Fine Biotech Co., China), hippocampal Nrf2 (catalogue no. MBS752046, MyBioSource, USA), hippocampal LC3-1 (catalogue no. ZC-58055, ZCIBIO Co., China), and hippocampal LC3-II (catalogue no. CBA-5116, Cell Biolabs Inc., USA).

### Spectrophotometrical analysis

Hippocampal MDA level was determined spectrophotometrically using (Catalog No. K739-100, BioVision assay kit, USA), according to the manufacturer’s instructions. Thiobarbituric acid and MDA react in an acidic environment (95 °C) to yield a thiobarbituric acid-reactive product that is pink in color and measured at 534 nm.

### Real-time reverse transcriptase polymerase chain reaction (RT-PCR) quantitative analysis

The RNeasy Mini kit (Cat. # 74104, Qiagen, Hilden, Germany) was used to extract total RNA from hippocampal specimens to evaluate the mRNA expression levels of ASCL-4 and GPX4. RNA concentration and purity were evaluated using a NanoDrop 2000 spectrophotometer (Thermo Fisher Scientific, Wilmington, DE, USA). Subsequently, 1 µg of total RNA was reverse-transcribed into cDNA using the RevertAid First Strand cDNA Synthesis Kit according to the manufacturer’s instructions. Quantitative real-time PCR was performed using a StepOne Real-Time PCR System (Applied Biosystems, CA, USA) and PowerUp™ SYBR™ Green Master Mix (Applied Biosystems, Thermo Fisher Scientific, USA). The PCR reaction mixture contained SYBR Green Master Mix, cDNA template, and gene-specific primers. The (2− ΔΔCT) approach was used to measure the relative expression according to (Livak and Schmittgen 2001). GAPDH was used to normalize the relative expression of the ASCL-4 and GPX4 genes. The primer sequences are displayed in Table 1.

**Table 1:** The primer sequences for quantitative RT-PCR

mRNA species	Primer sequence (5′–3′)
ACSL4	Forward: 5′-TCCAAGCCAGAAAACTCAAGC-3′ Reverse: 5′-GGTGTACATGACAATGGCCAT-3′
GPX4	Forward: 5′-CCTGGCTGGCACCATGT-3′ Reverse: 5′-CACACGCAACCCCTGTACTT-3′
GAPDH	Forward: 5′-TGGATTTGGACGCATTGGTC-3′ Reverse: 5′-TTTGCACTGGTACGTGTTGAT-3′

### Statistical analysis

The data were checked for normality by the Shapiro–Wilk normality test and expressed as means ± SD. The statistically significant difference was considered at *P* < 0.05. Data analyses were carried out using GraphPad Prism (version 9, GraphPad Software Inc., USA). The data related to MWM (acquisition phase) were analyzed by Two-Way ANOVA, followed by Tukey’s post hoc test, while the data of the probe test of MWM and other measurements were analyzed using One-Way ANOVA, followed by Tukey’s post hoc test for multiple comparisons.

## Results

### Effect of clemastine on cognitive function (MWM test) in acetic acid-induced UC in rats

#### Effect of clemastine on the acquisition phase

**Fig. 2:**
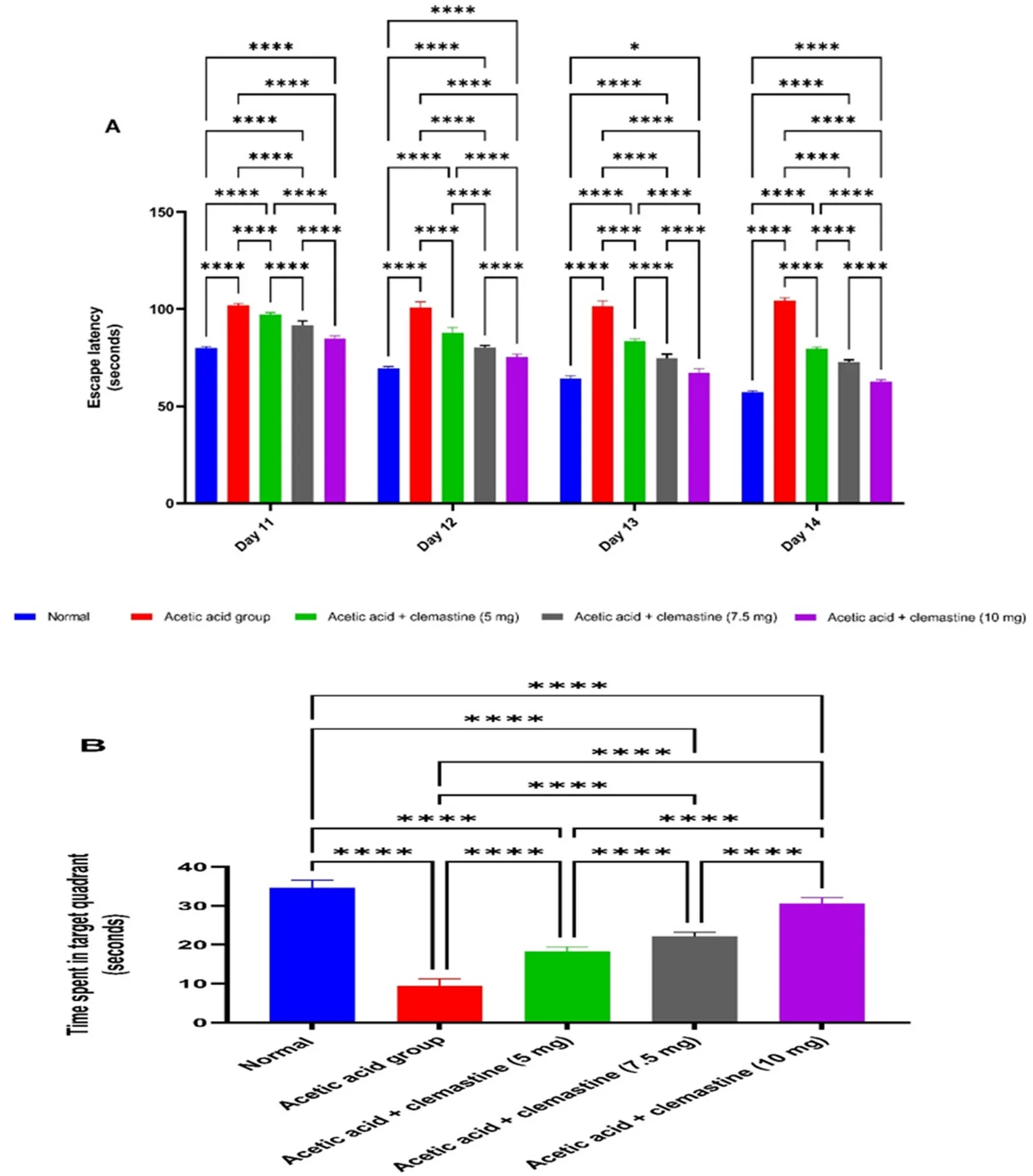
Effect of clemastine (5 or 7.5 or 10 mg/kg) on acetic acid-induced cognitive changes using MWM test. **A **escape latency during the acquisition phase and **B **the time spent in the target quadrant during the probe phase. Values are represented as mean (*n* = 10) ± SD. The escape latency was statistically analysed by two-way ANOVA followed by Tukey’s post hoc test for multiple comparisons, meanwhile the time spent in the target quadrant during the probe phase was statistically analysed by means of one-way ANOVA followed by Tukey’s post hoc analysis. The significance levels are as follows: *p < 0.05, ****p < 0.0001

The acetic acid group showed a significant increase in the mean of escape latency by 51% as compared to the normal group. The acetic acid + clemastine 5 mg group showed a significant decrease in the mean of escape latency by 15% as compared to the acetic acid group. Besides, the acetic acid + clemastine 7.5 mg group showed decrease in the mean of escape latency time by 22% and 8% as compared to the acetic acid group and acetic acid + clemastine 5 group, respectively. Finally, the acetic acid + clemastine 10 mg group showed significant decrease in the mean of escape latency by 29%, 17%, and 9% as compared to the acetic acid group, the acetic acid + clemastine 5 mg group, and the acetic acid + clemastine 7.5 mg group, respectively (Fig. 2A).

#### Effect of clemastine on the probe phase

The acetic acid group showed a dramatic decrease in the time spent in the target quadrant by 73% as compared to the normal group. The acetic acid + clemastine 5 mg group showed a significant increase in the time spent in the target quadrant by 94% as compared to the acetic acid group. On the other hand, the acetic acid + clemastine 7.5 mg showed increase in the time spent in the target quadrant by 140% and 21% as compared to the acetic acid group and the acetic acid + clemastine 5 group, respectively. Finally, the acetic acid + clemastine 10 mg group showed significant increase in the time spent in the target quadrant by 220%, 68%, and 38% as compared to the acetic acid group, the acetic acid + clemastine 5 mg group, and the acetic acid + clemastine 7.5 mg group, respectively (Fig. 2B).

### Effect of clemastine on colon and hippocampal histopathological changes in acetic acid-induced UC in rats

#### Effect of clemastine on colon histopathological changes

**Fig. 3:**
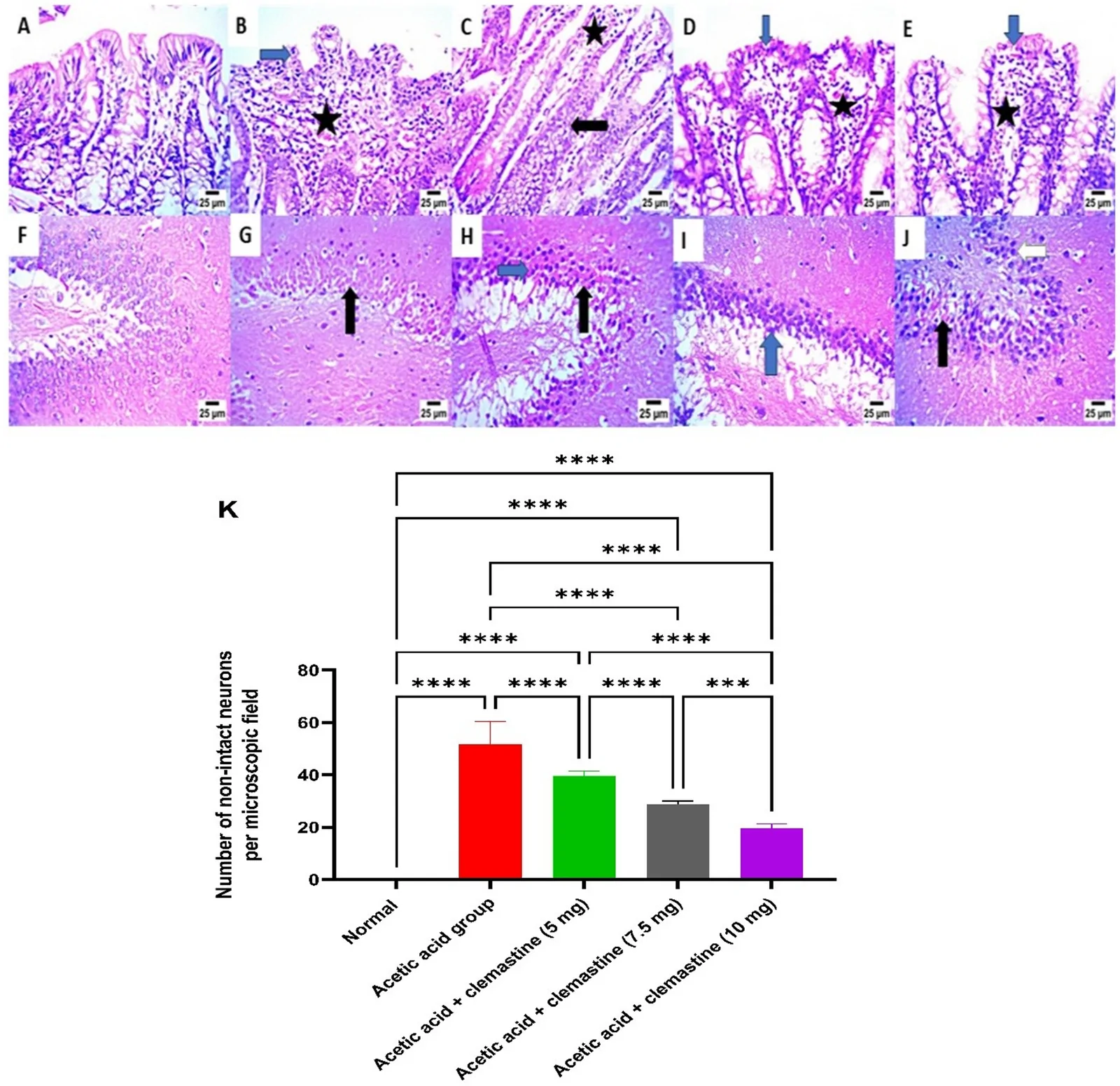
Effect of clemastine (5 or 7.5 or 10 mg/kg) on H&E with photomicrograph showing pathological changes in the colon and hippocampus. **A**–**E** Illustrative images showing the H&E-stained colon histological sections (scale bar: 25 μm). **A** normal histological structure of intestinal mucosa. **B** severe necrobiotic changes in enterocytes (blue arrow) accompanied by infiltration of intestinal mucosa by a high number of inflammatory cells mainly lymphocytes and eosinophils (star). **C** severe hyperplasia of enterocytes (black arrow) with infiltration of intestinal mucosa by inflammatory cells, mainly lymphocytes and eosinophils (star). **D** partial regeneration of intestinal epithelium (blue arrow) with infiltration of intestinal mucosa by moderate number of inflammatory cells mainly lymphocytes and eosinophils (star). **E** almost complete regeneration of intestinal epithelium (blue arrow) with infiltration of intestinal mucosa by few number of inflammatory cells mainly lymphocytes and eosinophils (star). **F**–**J** Illustrative images showing the H&E-stained histological sections of hippocampus (scale bar: 25 μm) **F** normal histological structure of neurons. **G** high number of necrotic neurons (black arrow). **H** moderate number of degenerated neurons with severe nuclear pyknosis (blue arrow) and moderate number of necrotic neurons (black arrow). **I** numerous neurons with nuclear pyknosis (blue arrow). **J** few number of necrotic neurons (black arrow). Data are expressed as mean ± SD of four animals of each group. Statistical analysis was carried out by using GraphPad Prism, One -Way ANOVA followed by Tukey’s post hoc test for multiple comparisons. The significance levels are as follows: ***p < 0.001, ****p < 0.0001

As demonstrated in Fig. 3A, the intestinal mucosa in the normal group had a normal histological structure. On the other hand, severe necrobiotic alterations in enterocytes were noted in the acetic acid-treated group with a large number of inflammatory cells infiltration in the mucosa, primarily lymphocytes and eosinophils (Fig. 3B), as compared to the normal group. The acetic acid + clemastine 5 mg group (Fig. 3C) displayed significant inflammatory cell infiltration, primarily lymphocytes and eosinophils, in the mucosa along with severe hyperplasia of enterocytes. On the other hand, partial intestinal epithelial regeneration and infiltration of a moderate number of inflammatory cells, primarily lymphocytes and eosinophils, were observed in the acetic acid + clemastine 7.5 mg group (Fig. 3D). In the acetic acid + clemastine 10 mg group, complete regeneration of the intestinal epithelium was detected with only a small number of inflammatory cells infiltration, primarily lymphocytes and eosinophils, in the mucosa (Fig. 3E).

#### Effect of clemastine on hippocampal histopathological alterations

The brain examination showed that the neurons in the normal group (Fig. 3F) had a normal histological structure. Necrotic neurons with a high number of non-intact neurons were detected in the hippocampus in the group treated with acetic acid (Fig. 3G) as compared to the normal group. A decrease in number of non-intact neurons by 23% with a moderate number of necrotic cells and degenerated neurons with severe pyknosis were observed in the acetic acid + clemastine 5 mg group (Fig. 3H), as compared to the acetic acid group. The acetic acid + clemastine 7.5 mg group (Fig. 3I) showed also reduction of non-intact neurons by 44% and 27% as compared to the acetic acid group and acetic acid + clemastine 5 group, respectively, with numerous neurons with nuclear pyknosis. On the other hand, the acetic acid + clemastine 10 mg group (Fig. 3J) demonstrated few necrotic neurons with decrease in number of non-intact neurons by 63%, 51%, and 32% as compared to the acetic group, the acetic acid + clemastine 5 mg group, and the acetic acid + clemastine 7.5 mg group, respectively.

### Effect of clemastine on the colon TNF-α and caspase-3 levels as well as serum TNF-α level in acetic acid-induced UC in rats

**Fig. 4:**
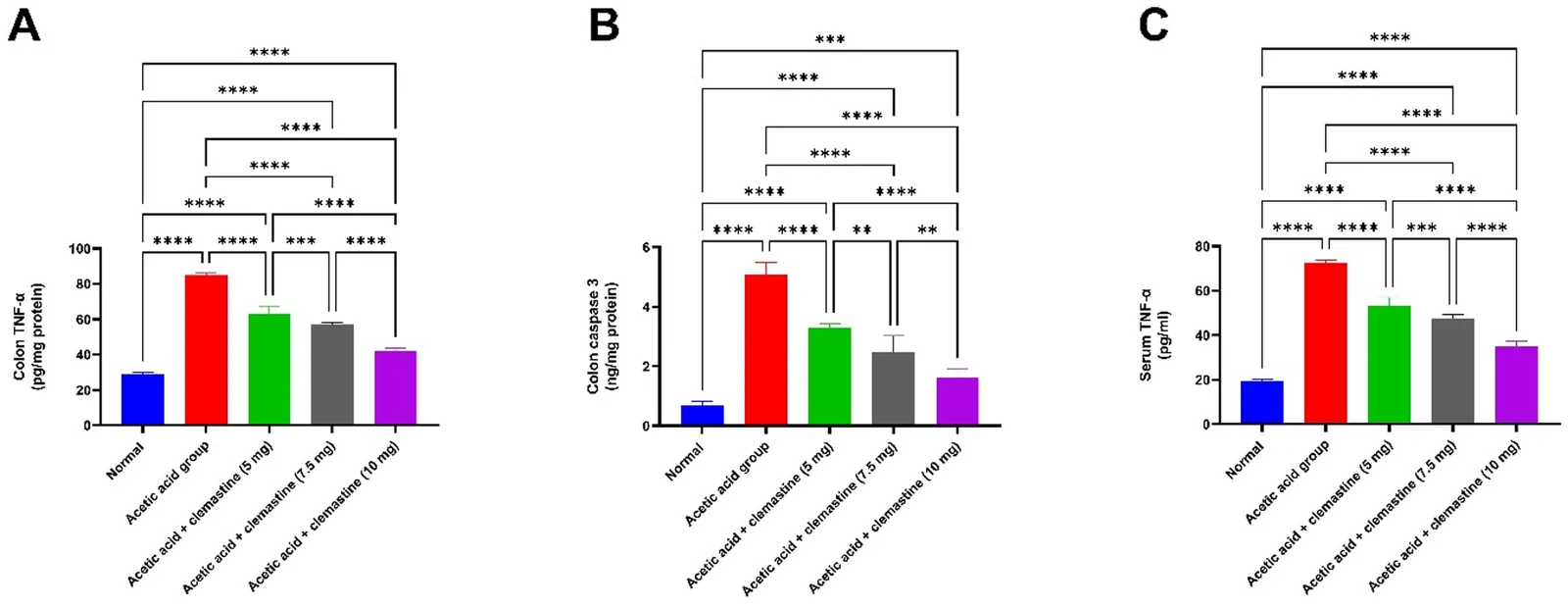
Effect of clemastine (5 or 7.5 or 10 mg/kg) on colon contents of (**A**); TNF-α, (**B**); caspase-3, and (**C**); serum TNF-α level. Values are represented as mean (*n* = 6) ± SD. Statistical analysis was done using one-way ANOVA followed by Tukey’s post hoc test The significance levels are as follows: **p < 0.01, ***p < 0.001, ****p < 0.0001

The obtained results demonstrated that the acetic acid group showed a significant increase in colonic contents of TNF-α and caspase-3 and serum level of TNF-α by 190%, 6.3-fold, and 270%, respectively, as compared to the normal group. The acetic acid + clemastine 5 mg group showed significant decrease in colon contents of TNF-α and caspase-3 and serum level of TNF-α by 26%, 35%, and 27% as compared to the acetic acid group, respectively. On the other hand, the acetic acid + clemastine 7.5 mg showed a decrease in colon levels of TNF-α, caspase-3, and serum levels of TNF-α by (33%, 51%, and 35%) as well as (10%, 24%, and 11%) as compared to the acetic acid group and acetic acid + clemastine 5 group, respectively. Interestingly, acetic acid + clemastine 10 mg group showed a significant decrease in colon levels of TNF-α, caspase-3, and serum levels of TNF-α by (50%, 69%, and 51%), (33%,51%, and 34%) as well as (26%, 36%, and 26%) as compared to the acetic acid group, the acetic acid + clemastine 5 mg group, and the acetic acid + clemastine 7.5 mg group, respectively (Fig. 4A, B, and C).

### Effect of clemastine on hippocampal AKT/PI3K/GSK-3β and oxidative stress parameters (Nrf2 and MDA) in acetic acid-induced UC in rats

**Fig. 5:**
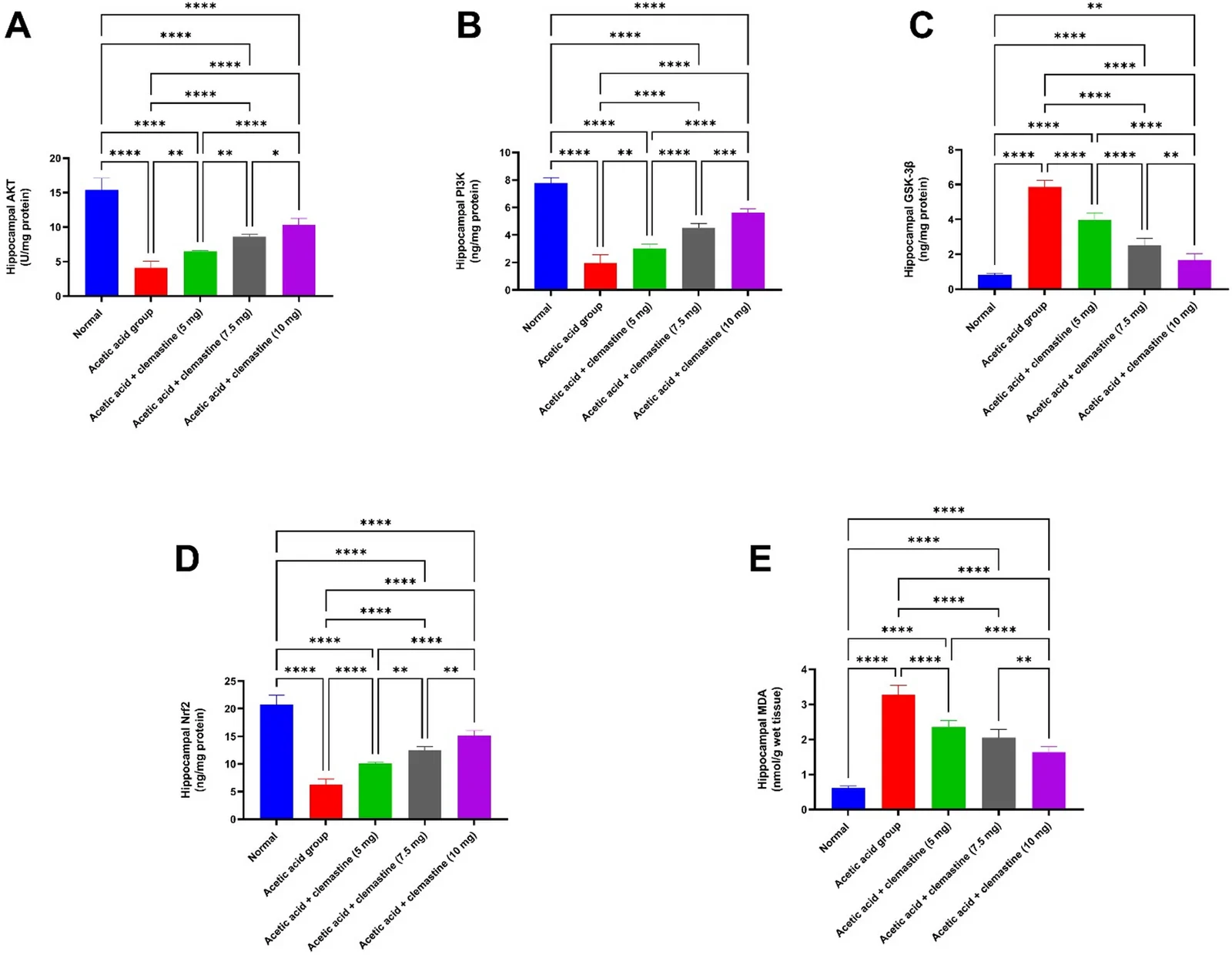
Effect of clemastine (5 or 7.5 or 10 mg/kg) on hippocampal (**A**); AKT, (**B**); PI3K, (**C**); GSK-3β, (**D**); Nrf2, and (**E**); MDA contents in rats with acetic acid-induced UC. Values are represented as mean (*n* = 6) ± SD. Statistical analysis was done using one-way ANOVA followed by Tukey’s post hoc test. The significance levels are as follows: *p < 0.05, **p < 0.01, ***p < 0.001, ****p < 0.0001

The present data reveal that the acetic acid group demonstrated significant decrease in levels of AKT, PI3K and Nrf2 by 73%, 75%, and 70%, respectively, along with elevation of the levels of GSK-3β and MDA by 6.3-fold and 4.3-fold, respectively, as compared to the normal group. The acetic acid + clemastine 5 mg group showed significant increase in levels of AKT, PI3K, and Nrf2 by 56%, 58%, and 63%, respectively along with significant reduction of the levels of GSK-3β and MDA by 33% and 28%, respectively, as compared to the acetic acid group. The acetic acid + clemastine 7.5 mg group revealed increase in levels of AKT, PI3K and Nrf2 by (110%, 140% and 110%) and (34%, 50%, and 24%), respectively, along with significant reduction of the levels of GSK-3β and MDA by (57% and 36%) and (34% and 9%), as compared to the acetic acid group and the acetic acid + clemastine 5 mg group, respectively. The acetic acid + clemastine 10 mg group demonstrated elevation of the levels of AKT, PI3K and Nrf2 by (150%, 190% and 140%), (61%, 87%, and 50%) and (20%, 24%, and 21%), respectively, along with significant reduction of the levels of GSK-3β and MDA by (72%, 59%, and 36%), and (50%, 30%, and 24%) as compared to the acetic acid group, the acetic acid + 5 mg group and the acetic acid + 7.5 mg group, respectively (Fig. 5A, B, C, D and E).

### Effect of clemastine on hippocampal autophagic (LC3-I and LC3-II) and ferroptotic markers (ASCL-4 and GPX4) in acetic acid-induced UC in rats

**Fig. 6:**
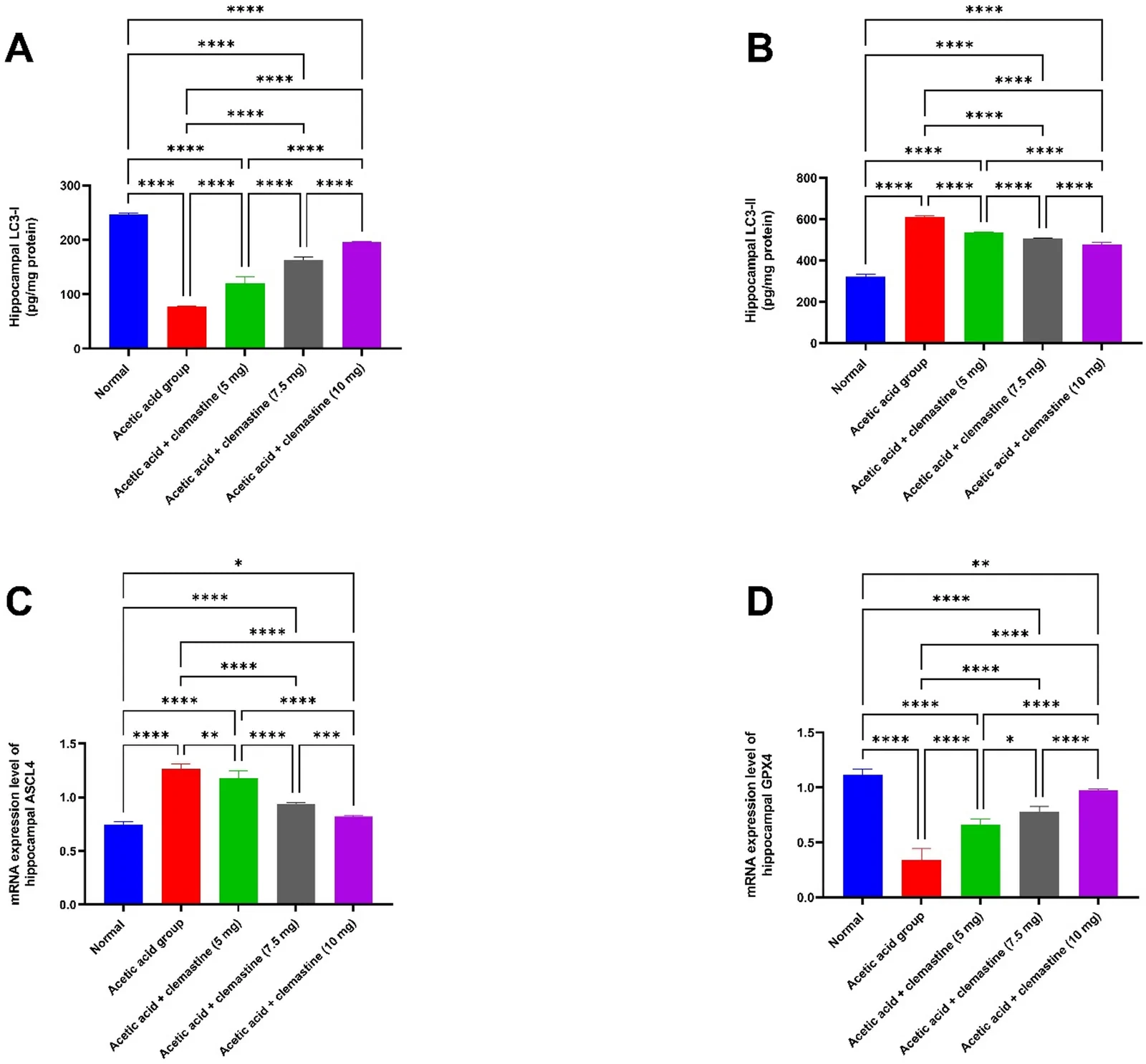
Effect of clemastine (5 or 7.5 or 10 mg/kg) on hippocampal contents of (**A**); LC3-I and (**B**); LC3-II, as well as mRNA expression of (**C**); ASCL-4, and (**D**); GPX4 in rats with acetic acid-induced UC. Values are represented as mean (*n* = 6) ± SD. Statistical analysis was done using one-way ANOVA followed by Tukey’s post hoc test. The significance levels are as follows: *p < 0.05, **p < 0.01, ***p < 0.001, ****p < 0.0001

The current results clarify that the acetic acid group showed significant decrease in level of LC3-1 and mRNA expression of GPX4 by 69% and 69%, respectively, along with elevation in level of LC3-II and mRNA expression of ACSL4 by 90% and 70%, as compared to the normal group, respectively. The acetic acid + clemastine 5 mg group revealed significant increase in level of LC3-1 and expression of GPX4 by 56% and 94%, along with significant reduction of level of LC3-II and expression of ACSL4 by 12% and 7% as compared to the acetic acid group, respectively. On the other side, the acetic acid + clemastine 7.5 mg group demonstrated significant increase in level of LC3-1 and expression of GPX4 by (110% and 130%) and (36% and 17%), along with significant reduction of level of LC3-II and expression of ACSL4 by (17% and 26%) and (5% and 18%) as compared to the acetic acid group and the acetic acid + clemastine 5 mg group, respectively. The acetic acid + clemastine 10 mg group showed substantial increase in level of LC3-1 and mRNA expression of GPX4 by (150% and 180%), (63% and 47%), as well as (20% and 26%) besides significant reduction of level of LC3-II and mRNA expression of ACSL4 by (21% and 34%), (10% and 27%), as well as (5% and 11%), as compared to the acetic acid group, the acetic acid + 5 mg group, and the acetic acid group, the acetic acid + 7.5 mg group, respectively (Fig. 6A, B, C, and D).

### Effect of clemastine on hippocampal Aβ, tau protein, and Iba-1, as well as colonic claudin-1 immunoexpressions in acetic acid-induced UC in rats

**Fig. 7:**
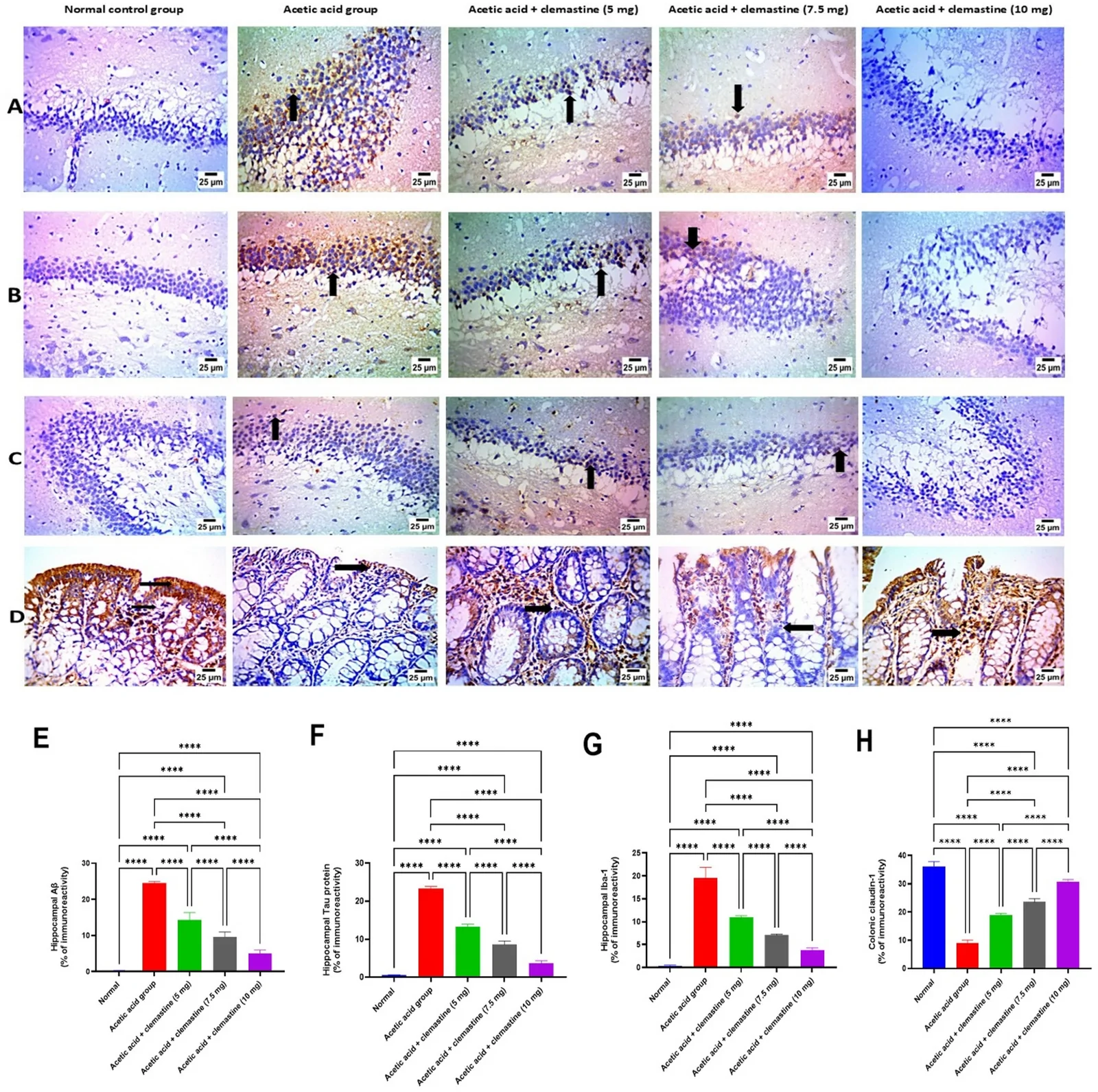
Effect of clemastine (5 or 7.5 or 10 mg/kg) on IHC evaluation of hippocampal (**A**); Aβ, (**B**); tau protein, (**C**); Iba-1, as well as (**D**); colonic claudin-1 in rats with acetic acid-induced UC (scale bar: 25 μm). (**E**), (**F**), (**G**), and (**H**) represent analyses of images of Aβ, tau, Iba-1, and claudin-1 immunoreactivity (% area), respectively. Values are represented as mean (*n* = 4) ± SD. Statistical analysis was done using one-way ANOVA followed by Tukey’s post hoc test The significance levels are as follows: ****p < 0.0001

The photographs of IHC demonstrated the acetic group with a marked increase in the percentage of area of positive reaction by 101-fold, 46-fold, and 52-fold for Aβ, tau protein, and Iba-1, respectively along with a significant reduction of colon claudin-1 reaction area by 75% as compared to the normal group. The acetic acid + clemastine 5 mg group showed a significant decrease in the percentage of reaction area by 42%, 43%, and 44% for Aβ, tau protein, and Iba-1, respectively, along with an increase in colon claudin-1 positive reaction area % by 110% as compared to the acetic group. On the other hand, the acetic acid + clemastine 7.5 mg showed a significant decrease in the reaction area % by 61%, 63%, and 64% as well as 33%, 35%, and 35% for Aβ, tau protein, and Iba-1, respectively along with an increase in expression of claudin-1 by 160% and 26% as compared to the acetic group and the acetic acid + clemastine 5 mg group, respectively. The acetic acid + clemastine 10 mg revealed a marked attenuation of the reaction area % by (79%, 84%, and 81%), (64%, 72%, and 65%), and (47%, 57%, and 46%) for Aβ, tau protein, and Iba-1, respectively along with an elevation in colon claudin-1 reaction area % by 240%, 63%, and 30% as compared to the acetic group, the acetic acid + clemastine 5 mg group, the acetic acid + clemastine 7.5 mg group, respectively (Fig. 7A–H).

## Discussion

UC is increasingly recognized to involve not only the gut but also to have significant effects on the brain, notably through impairing cognitive function (de Sire et al. 2024; Elegezy et al. 2025). The findings of the present study identify a complex interplay between colonic inflammation and cognitive impairment through affecting PI3K/Akt/GSK-3 signaling, ferroptosis, and autophagy, with a clear role for clemastine as a promising multi-targeted neuroprotective agent. As demonstrated in previous studies, the key features of UC are peripheral inflammation, dysregulated mucosal immunity, and overproduction of cytokines such as TNF-α and IL-6 that amplify local and systemic inflammatory responses (Tang et al. 2025; Bu et al. 2025). Locally in the colon, these mediators disrupt the epithelial barrier through alteration of tight junction proteins, with depletion of the protective mucosal layer. This leaky gut barrier permits translocation of bacterial products, leading to sustained immune activation in the lamina propria and spilling inflammatory signals into the circulation, thus linking intestinal pathology to extra-intestinal manifestations of UC (Peng et al. 2024; Tang et al. 2025; Tamer et al. 2025). In the current model, acetic acid-induced UC showed severe colonic damage, heavy inflammatory cell infiltration, and significant increase in colon TNF-α and caspase-3 and serum TNF-α, along with decreased claudin-1 expression, indicating epithelial barrier leakage and systemic inflammatory spillover.

Clemastine, specifically at a dose of 10 mg/kg/day, significantly reduced colonic and systemic inflammation (TNF-α) with decreased caspase-3 content in colon, improved mucosal architecture, and upregulated claudin-1 expression, indicating reinforcement of tight junction and barrier function (Mike et al. 2023). The preservation of gut integrity is consistent with reports demonstrating that barrier repair and cytokine suppression in experimental UC could reduce bacterial translocation, systemic inflammation, and injury to distant organs, including the brain (Soranno et al. 2025; Oami et al. 2025). Similarly, clemastine has revealed peripheral anti-inflammatory actions and protection of gut-barrier properties in other neuroinflammatory and demyelinating models, supporting the current findings of how peripheral modulation could be attained by reshaping of the gut–brain axis (Johansen et al. 2011; Su et al. 2018; Abdelnaser et al. 2025). By dampening colonic cytokine surges and improving the barrier function, clemastine could limit the systemic inflammatory condition (Abdelnaser et al. 2025), which is the hallmark of active UC. Importantly, systemic inflammation is a key link in the gut-brain axis that underpins extra-intestinal complications, with neurodegeneration and cognitive impairment being among those most consistently reported both clinically and experimentally in UC (Günther et al. 2021; Camberos-Barraza et al. 2024).

Chronic systemic inflammation directly affects BBB integrity, allowing leakage of systemic inflammatory mediators and leading to disturbance in major intracellular pro-survival pathways like PI3K/Akt/GSK-3β axis (Acosta-Martinez and Cabail 2022). The current data demonstrate that acetic acid-induced UC was associated with marked declines in both PI3K and Akt, and a remarkable increase in GSK-3β expression within the hippocampus, accompanied by elevated number of necrotic neurons. These results were associated with increased MDA and reduced Nrf2 contents together with upregulated Iba-1 immunoreactivity, indicative of increased oxidative stress and microglial activation as demonstrated by previous reports (Kramer et al. 2009; Kumar et al. 2025). Unconstrained GSK-3β activity accelerates the shift toward pro-apoptotic and pro-inflammatory gene expression, increasing neuronal damage, which is commonly seen in models of inflammatory bowel disease and neurodegenerative diseases (Lee et al. 2022). Increased GSK-3β not only promotes neuroinflammation but also reduces neurogenesis and synaptic plasticity, thus promoting cognitive decline (Yu et al. 2023). Overactivation of microglia, which is evidenced in the current model by the elevated expression of Iba-1, also could establish a self-amplifying loop between inflammation and neuronal damage, implicating the role of sustained microglial activation as a core mechanism for long-term cognitive dysfunction in inflammatory bowel disease and other chronic inflammatory disorders (Fornari Laurindo et al. 2023). Specifically, the high dose of clemastine (10 mg/kg/day) demonstrated normalization of PI3K/Akt/GSK-3β axis, with upregulation of PI3K and Akt and reduction of GSK-3β, complemented by harmonized improvements of oxidative stress markers, microglial activation, and overall neuronal survival which are in parallel with previous studies demonstrating the neuroprotective effects of clemastine on PI3K/AKT/GSK-3β axis (Yuan et al. 2020; Zuo et al. 2023; Badawi et al. 2024). These findings underpin the therapeutic potential of clemastine as a modulator of PI3K/Akt/GSK-3β signaling in neuronal inflammation, consistent with similar protective dynamics reported in models of stroke, neurodegeneration, and IBD-related brain injury (Rivera et al. 2022; Jiang et al. 2023b).

In the present work, the neuroprotective effects of clemastine also included suppression of microglial activation and hippocampal Iba-1 immunoreactivity, as well as enhancement of cellular antioxidant capacity, as demonstrated by increased Nrf2 and decreased MDA levels. These neuroprotective effects of clemastine have been similarly shown in experimental models of autoimmune encephalomyelitis and other neurological disorders, where it mitigates toxicity from microglial activation (Zhi et al. 2021; Jiang et al. 2023a).

Evidence also indicates that ferroptosis, a regulated form of cell death that is iron-dependent and characterized by increased lipid peroxidation, is a pivotal mechanism driving neuronal damage in UC due to chronic inflammation and impaired autophagy (Costa et al. 2023; Feng et al. 2023). In the current model, the UC group revealed decreased hippocampal GPX4 (a ferroptosis inhibitor) and increased ACSL4 (a ferroptosis driver) together with reduced LC3-I and elevated LC3-II levels, indicating strong ferroptotic drive and impaired autophagic signaling. This pathological pattern is in agreement with recent research showing that ferroptosis is turned on upon UC flares in both colonic and brain tissues, promoting neuronal damage through the gut-brain axis (Ru et al. 2024; Zhou et al. 2025). Ferroptosis is closely linked to impaired autophagy through disruption of autophagic flux, contributing to the accumulation of damaged mitochondria and lipid peroxides and aggravation of neuronal death (Liu et al. 2025). Importantly, clemastine administration reversed this impaired ferroptotic/autophagic signaling in a dose-dependent fashion, as demonstrated by increased GPX4 and LC3-I levels and decreased ACSL4 and LC3-II levels in addition to normalization of neuronal histology. Thus, targeting either ferroptosis or impaired autophagy can significantly reduce the severity of experimentally-induced UC and limit its sequelae within the CNS (Yang et al. 2022; Zhang et al. 2023).

Obviously, patients with UC typically present with disturbances in memory, attention, and executive function, which correlate with disease flares (Al Balakosy et al. 2024). Functional neuroimaging has shown that these disturbances are closely related to altered activity in the hippocampus and posterior cingulate cortex, all structures that are highly sensitive to inflammatory signaling and oxidative stress (Wang et al. 2022). Clinical findings of hippocampal-dependent cognitive impairment are closely mimicked by the behavioral deficits in the MWM experiment, as evidenced herein by increased escape latency and reduced target quadrant time, providing strong evidence that chronic colonic inflammation could result in hippocampal-dependent cognitive impairment (Fan et al. 2019). Additionally, our current findings reveal that chronic inflammation in UC could induce Alzheimer-like pathology in the brain, as demonstrated by the elevated hippocampal Aβ and tau immunoreactivity, which complements the established interconnection between overactive GSK-3β and chronic inflammation on one side and the promotion of the amyloidogenic pathway of amyloid precursor protein processing and tau phosphorylation on the other side. Clinical studies in patients with inflammatory bowel disease have reported these molecular changes with an increased risk of dementia (Cui et al. 2022; Garmendia et al. 2023). Interestingly, clemastine (10 mg/kg/day) almost completely inhibited both Aβ and tau accumulation in the current investigation, with normalization of behavioral alterations observed in the UC group. By reducing colonic inflammation and apoptosis and improving the gut barrier function, clemastine could limit the upstream inflammatory signaling that would feed CNS pathology.

### Study limitations and future perspectives

Future studies are required to examine the protective effects of clemastine in other models of chronic colitis like those chemically-induced using dextran sodium sulfate or trinitrobenzene sulfonic acid. Additionally, using a relatively longer treatment period could help better evaluation of the effect of clemastine on brain-gut axis dynamics as well as the impact of sustained systemic inflammation on cognitive function.

## Conclusion

The present findings reveal that UC-induced systemic inflammation sets off a series of harmful neuroinflammatory outcomes that could drive neuronal damage and cognitive impairment. The current data also support the role of clemastine as a valuable agent for the management of UC-associated brain disorders through addressing peripheral gut and systemic inflammation with beneficial effects on the gut-brain axis. Additionally, clemastine reveals central effects through recalibrating PI3K/Akt/GSK-3β signaling with beneficial outcomes on oxidative stress, ferroptosis, microglial overactivation, impaired autophagic signaling, and thus elimination of the harmful neurodegenerative protein aggregates. The successful disruption of this peripheral pathogenic signaling provides a well-defined approach for reducing the cognitive impairment linked to UC and other systemic inflammatory-driven disorders.

## Data Availability

The data that support the findings of this study are available from the corresponding author upon reasonable request.

## Abbreviations

Aβ: Amyloid beta
ACSL4: Acyl-CoA synthetase long chain family member 4
Akt: Phosphokinase B
ANOVA: Analysis of variance
BBB: Blood-brain barrier
CNS: Central nervous system
DAB: Diaminobenzidine
ELISA: Enzyme-linked immunosorbent assay
GPX4: Glutathione peroxidase 4
GSK-3β: Glycogen synthase kinase-3beta
H&E: Hematoxylin and eosin
Iba-1: Ionized calcium-binding adapter molecule 1 (Microglial activation marker)
IHC: Immunohistochemistry
IL-1β: Interleukin 1 beta
LC3-I: Microtubule-associated protein 1 light chain 3-I
LC3-II: Microtubule-associated protein 1 light chain 3-II
MDA: Malondialdehyde
MWM: Morris water maze
Nrf2: Nuclear factor-related factor 2
PI3K: Phosphatidylinositol-3-kinase
RT-PCR: Real-time polymerase chain reaction
TNF-α: Tumor necrosis factor-alpha
UC: Ulcerative colitis
